# Maternal adverse childhood experiences and behavioral problems in preschool offspring: the mediation role of parenting styles

**DOI:** 10.1186/s13034-023-00646-3

**Published:** 2023-08-10

**Authors:** Shengyu Luo, Dezhong Chen, Chunrong Li, Li Lin, Weiqing Chen, Yan Ren, Yuchi Zhang, Fenglin Xing, Vivian Yawei Guo

**Affiliations:** 1 Department of Epidemiology, School of Public Health, Sun Yat-sen University, Guangzhou, Guangdong China; 2 School of Medicine, Chengdu Women’s and Children’s Central Hospital, University of Electronic Science and Technology of China, Chengdu, Sichuan China; 3 Chengdu Jintang County Maternal and Child Health Hospital, Chengdu, Sichuan China; 4 Chengdu Qingyang District Maternal and Child Health Hospital, Chengdu, Sichuan China

**Keywords:** Adverse childhood experiences, Intergeneration, Behavioral problems, Parenting styles, Preschool children, Mediation

## Abstract

**Background:**

Maternal history of adverse childhood experiences (ACEs) has been found to be associated with children’s health outcomes. However, the underlying mechanisms were unclear. This study aimed to examine the association between maternal ACEs and behavioral problems in their preschool offspring and to explore the potential mediating role of maternal parenting styles in the association.

**Methods:**

A cross-sectional study was conducted involving 4243 mother-child dyads in Chengdu, China. Mothers completed the Adverse Childhood Experiences-International Questionnaire (ACE-IQ) to assess their history of ACEs (i.e., physical abuse, emotional abuse, physical neglect, emotional neglect, witnessing domestic violence, household substance abuse, household mental illness, incarcerated household member, parental separation or divorce, parental death, bullying, and community violence), the short Egna Minnen Beträffande Uppfostran Parent Form (S-EMBU-P) to evaluate their parenting styles (i.e., emotional warmth, rejection, and overprotection), and the 48-item Conners’ Parent Rating Scale (CPRS-48) to measure behavioral problems in their children. Logistic regression models were established to examine the association between cumulative number of maternal ACEs and children’s behavioral problems. The mediating role of parenting styles in this association was explored by generalized structural equation models (GSEM).

**Results:**

Of the participating mothers, 85.8% (n = 3641) reported having experienced at least one type of ACE. Children of mothers with ≥2 ACEs showed a significantly increased risk of behavioral problems across all dimensions, including conduct problems, learning problems, psychosomatic problems, impulsive-hyperactive, anxiety, and hyperactivity index, in both crude and adjusted models (all *p*-values < 0.05). Dose-response patterns were also observed between the cumulative number of maternal ACEs and children’s behavioral problems. In addition, maternal parenting styles of rejection emerged as a significant mediator, accounting for approximately 8.4–15.0% of the associations.

**Conclusions:**

Our findings indicated an intergenerational association of maternal ACEs with behavioral problems in preschool offspring, which was mediated by maternal parenting styles of rejection. Early screening and targeted intervention strategies are critical to mitigate the downstream consequences of maternal ACEs on young children’s outcomes. Providing support and resources to improve parenting skills may prove beneficial.

**Supplementary Information:**

The online version contains supplementary material available at 10.1186/s13034-023-00646-3.

## Background

Adverse childhood experiences (ACEs) include various forms of traumatic or stressful events that occurred during the early years of life [1]. These experiences range from maltreatment of neglect and abuse to household dysfunctions, such as witnessing domestic violence or growing up in the absence of parents, as well as living in an unsafe community [2]. Cumulative studies have documented a significant association between exposure to ACEs and impairment in an individual’s physical, emotional, and cognitive development during adolescence [3–5]. These detrimental effects may persist into adulthood, leading to increased risk of mental and physical diseases later in life [6–8].

In recent years, emerging research has also highlighted the intergenerational transmission of ACEs, establishing an association between maternal ACEs and children’s behavioral and health outcomes [9–11]. For example, a cross-sectional study of 4243 mother-child dyads has indicated that preschool children with maternal ACE exposure tended to have poorer health-related quality of life [9]. In addition, a US-based cross-sectional study has revealed a positive association between mothers experiencing four or more ACEs and children’s poor behavioral outcomes [10]. Similarly, another cross-sectional study conducted in China has also found that maternal ACEs was linked to increased risk of behaviors problems in their preschool offspring [11].

The mechanisms underlying the link between maternal ACEs and children’s outcomes have not been fully elucidated. One possible explanation is related to parenting styles, a multifaced factor that has been found to be closely associated with children’s physical, psychological, and social development [12]. Parenting styles refer to how parents support, communicate, discipline, and supervise their children, which include two primary dimensions, i.e., warmth and strictness [13]. Previous studies have shown that mothers who had experienced adversities during childhood were more likely to adopt problematic parenting styles towards their children, such as low parental sensitivity, difficulty in setting boundaries, and high aggressiveness [14, 15], which in turn could increase the risk of their children developing behavioral problems [16]. Furthermore, parenting styles in China differ from those in other cultures [17, 18], potentially yielding a different association with behavioral problems in their children [19]. Nevertheless, studies exploring the role of parenting styles in the association between maternal ACEs and children’s behaviors have produced mixed findings and have predominantly been conducted in North America and Europe [20–24]. Based on the aforementioned evidence, we hypothesized that parenting styles might serve as a potential mediator in the association between maternal exposure to ACEs and behavioral problems in Chinese preschool children.

Therefore, this study aimed to replicate the findings from previous research that demonstrated an association between maternal ACEs and behavioral problems in their preschool children. The possible mediating role of maternal parenting styles in this association was also evaluated in the context of Chinese culture.

## Methods

### Study design and participants

This cross-sectional study was conducted from May to July 2021 in Chengdu, a megacity located in western China. It consists of 12 urban districts, 5 county-level cities, and 3 counties. To select representative children from preschools, we adopted a multistage sampling strategy. First, 4 urban districts, 2 county-level cities, and 1 county were randomly selected. Second, two preschools were further randomly chosen from each selected area, with 14 preschools in total. Last, all children in the selected preschools and their parents were invited to join the study. In total, caregivers of 5102 preschool children have finished an online questionnaire (response rate: 86.5%). We have further excluded 795 children as the questionnaires were answered by their fathers, 23 children with answers from their grandparents or other caregivers, and 41 children with misreport of age, leaving 4243 mother-child dyads in the current analysis.

Ethical approval for the study was obtained from Sun Yat-sen University (Reference number: 2021[116]), and informed consent was obtained from each parent prior to participation.

### Measurement of maternal ACEs

Maternal ACE exposure was measured by the Adverse Childhood Experiences-International Questionnaire (ACE-IQ), which has been validated in Chinese population [25]. It consists of 29 items covering childhood adversities of abuse, neglect, household challenges, and exposure to community and collective violence. We excluded four items of sexual abuse given the sensitivity of it in Chinese culture and four items of collective violence as it was uncommon in China. The final questionnaire was comprised of 21 items measuring 12 types of ACEs, i.e., physical abuse (2 items), emotional abuse (2 items), physical neglect (3 items), emotional neglect (2 items), witnessing domestic violence (3 items), household substance abuse (1 item), household mental illness (1 item), incarcerated household member (1 item), parental separation or divorce (1 item), parental death (1 item), bullying (1 item), and community violence (3 items). Table S1 [see Additional file 1] provides a list of the detailed questionnaire items and definitions of positive answers to each ACE category. Mothers who reported a positive answer to any item within a specific ACE category were considered exposed and given a code of 1 for that category. Cumulative ACE scores ranging from 0 to 12 were then determined by adding up the scores for each of the 12 types of ACEs. These scores were then binned into 5 categories: (1) 0 ACE, (2) 1 ACE, (3) 2 ACEs, (4) 3 ACEs, and (5) ≥4 ACEs.

### Measurement of maternal parenting styles

Mothers self-reported their parenting styles by the short Egna Minnen Beträffande Uppfostran Parent Form (S-EMBU-P), a 21-item questionnaire that measures three dimensions, including emotional warmth (7 items), rejection (6 items), and overprotection (8 items) [26]. The questionnaire has been validated in Chinese population and the reliability coefficient was 0.93 in the current study [27]. All questionnaire items were rated on a 4-point Likert scale (1 = never, 2 = seldom, 3 = often, and 4 = most of the time). To generate a score for each parenting style, the scores of all items within the corresponding dimension were summed. A higher score indicated greater levels of emotional warmth, rejective behaviors, and overprotection from mothers, respectively.

### Measurement of children’s behavioral problems

Children’s behavioral problems were reported by their mothers using the 48-item Conners’ Parent Rating Scale (CPRS-48), which was demonstrated to be valid in Chinese children [28]. It includes five dimensions of behavioral problems, i.e., conduct problems (12 items), learning problems (4 items), psychosomatic problems (5 items), impulsive-hyperactive (4 items), and anxiety (4 items). In addition, a hyperactivity index was constructed with 10 items that are considered to be the most sensitive to the effects of treatment [29]. Each item was rated on a 4-point Likert scale (0 = never, 1 = occasionally, 2 = often, and 3 = very often). The severity of each behavioral problem was represented by the average score of all items in the corresponding dimension. For each dimension, children with a score ≥90th percentile were considered as having symptoms of problematic behaviors [30].

### Covariates

#### Mothers

Mothers self-reported their age, current marital status (1 = married and 2 = unmarried), educational level (1 = junior high school or below, 2 = senior high school, and 3 = bachelor’s degree or above), monthly per-capita income (1 = 5000 RMB or below, 2 = 5001 ~ 10,000 RMB, 3 = 10,001 RMB or above, and 4 = uncertain, where 1 US$ ≈ 6.92 RMB), and negative emotional states over the past week.

Negative emotional states of depression, anxiety, and stress over the past week were measured with the Depression Anxiety Stress Scale-21 (DASS-21), which has been validated in Chinese population [31]. Each type of negative emotional state contains seven 4-point Likert items (0 = did not apply to me at all, 1 = applied to me to some degree, or some of the time, 2 = applied to me a considerable degree, or a good part of the time, 3 = applied to me very much, or most of the time) and a summary score can be further generated respectively [32]. To define negative emotional states, cutoff points of 9, 7, and 14 were used for depression, anxiety, and stress, respectively.

#### Children

Mothers reported their children’s age, gender (1 = boys, 2 = girls), whether the children were the only child in the family (0 = no and 1 = yes), and who their primary caregivers were (1 = mothers, 2 = fathers, and 3 = others).

### Statistical analysis

Maternal and children’s characteristics were presented as mean (SD) for continuous variables and frequency (percentage) for categorical variables. One-way analysis of variance (ANOVA) and Chi-square tests were applied to compare continuous and categorical variables by maternal ACE groups, respectively. To explore the dose-response patterns of characteristics across different maternal ACE groups, we performed polynomial contrasts of ANOVA trend tests for continuous variables and Mantel-Haenszel statistic for categorical variables.

The associations between maternal ACE exposure and behavioral problems in offspring were examined by logistic regression models. We first established crude models, and then controlled for maternal age, current marital status, educational level, monthly per-capita income, negative emotional states, as well as children’s age, gender, single-child status, and primary caregivers in the adjusted models. The results were presented as odds ratios (OR) and its 95% confidential intervals (CIs). Trend tests were also performed to portray dose-response patterns.

To evaluate the mediation role of maternal parenting styles, generalized structural equation models (GSEM) were applied with adjustment for aforementioned covariates. Unstandardized estimated coefficients were presented for direct, indirect, and total effects. The bootstrapping procedure with 1000 samples was further performed to obtain the bias-corrected 95% CI.

All statistical analyses were performed using Stata/SE 17.0 (StataCorp, College Station, TX). A two-tailed *p* < 0.05 was considered statistically significant.

## Results

Of the 4243 mothers included, 85.8% (n = 3641) experienced at least one ACE and 13.7% (n = 579) were exposed to four or more ACEs. Table 1 displays the comparison of maternal and children’s characteristics by maternal ACE groups. Compared to mothers without any ACE exposure, those experienced four or more ACEs were more likely to report unmarried status and negative emotional states. In terms of parenting styles, mothers with four or more ACEs reported lower level of emotional warmth and higher level of rejection and overprotection than their counterparts without history of ACEs. Of the 4243 children included, the mean age was 4.6 (SD = 1.0) years and 51.7% (n = 2193) were boys. Children of mothers who were exposed to a greater number of ACEs were more likely to be the only child in the family. In addition, higher prevalence of children’s behavioral problems was observed in all dimensions when the number of maternal ACEs increased.

**Table 1:** Maternal and children’s characteristics stratified by maternal ACE groups

		Number of ACEs					*p*-value for difference	*p*-value for trend
		0	1	2	3	≥4		
** N (%)**		602 (14.2)	1919 (45.2)	774 (18.2)	369 (8.7)	579 (13.7)		
**Maternal characteristics**								
Age (years), mean (SD)		33.7 (4.6)	33.0 (4.5)	32.9 (4.6)	33.2 (4.8)	33.3 (4.6)	0.022	0.298
Current marital status, n (%)							0.012	< 0.001
	Married	588 (97.7)	1855 (96.7)	750 (96.9)	351 (95.1)	546 (94.3)		
	Unmarried^a^	14 (2.3)	64 (3.3)	24 (3.1)	18 (4.9)	33 (5.7)		
Educational level, n (%)							< 0.001	0.840
	Junior high school or below	23 (3.8)	205 (10.7)	80 (10.3)	36 (9.8)	43 (7.4)		
	Senior high school	108 (17.9)	485 (25.3)	197 (25.5)	89 (24.2)	109 (18.8)		
	Bachelor’s degree or above	471 (78.2)	1226 (64.0)	497 (64.2)	243 (66.0)	427 (73.7)		
Monthly per-capita income, n (%)							< 0.001	0.725
	≤5000 RMB^b^	135 (22.4)	569 (29.7)	209 (27.0)	114 (30.9)	148 (25.6)		
	5001 ~ 10,000 RMB	170 (28.2)	492 (25.6)	200 (25.8)	90 (24.4)	154 (26.6)		
	≥10,001 RMB	240 (39.9)	587 (30.6)	261 (33.7)	127 (34.4)	214 (37.0)		
	Uncertain	57 (9.5)	271 (14.1)	104 (13.4)	38 (10.3)	63 (10.9)		
Negative emotional states, n (%)								
	Depression	3 (0.5)	59 (3.1)	35 (4.5)	33 (8.9)	88 (15.2)	< 0.001	< 0.001
	Anxiety	7 (1.2)	91 (4.7)	44 (5.7)	45 (12.2)	107 (18.5)	< 0.001	< 0.001
	Stress	6 (1.0)	64 (3.3)	39 (5.0)	41 (11.1)	94 (16.2)	< 0.001	< 0.001
Parenting styles, mean (SD)								
	Emotional warmth	24.6 (3.3)	22.2 (4.8)	22.4 (4.5)	22.7 (4.1)	23.1 (3.9)	< 0.001	< 0.001
	Rejection	7.0 (1.2)	7.5 (1.7)	7.7 (1.7)	7.9 (1.8)	8.1 (1.9)	< 0.001	< 0.001
	Overprotection	15.5 (2.9)	15.5 (2.9)	15.7 (2.9)	15.8 (2.7)	15.9 (2.7)	0.027	0.004
**Children’s characteristics**								
Age (years), mean (SD)		4.7 (1.0)	4.6 (1.0)	4.6 (1.0)	4.6 (1.0)	4.6 (1.0)	0.308	0.345
Gender, n (%)							0.498	0.133
	Boys	320 (53.2)	1012 (52.7)	385 (49.7)	185 (50.1)	291 (50.3)		
	Girls	282 (46.8)	907 (47.3)	389 (50.3)	184 (49.9)	288 (49.7)		
Single-child status, n (%)							0.001	< 0.001
	No	330 (54.8)	1082 (56.4)	419 (54.1)	181 (49.1)	274 (47.3)		
	Yes	272 (45.2)	837 (43.6)	355 (45.9)	188 (50.9)	305 (52.7)		
Primary caregivers, n (%)							0.130	0.004
	Mother	470 (78.1)	1426 (74.3)	571 (73.8)	255 (69.1)	415 (71.7)		
	Father	18 (3.0)	69 (3.6)	23 (3.0)	16 (4.3)	21 (3.6)		
	Others	114 (18.9)	424 (22.1)	180 (23.3)	98 (26.6)	143 (24.7)		
Conduct problems, n (%)							< 0.001	< 0.001
	No symptoms	589 (97.8)	1813 (94.5)	717 (92.6)	323 (87.5)	486 (83.9)		
	Symptoms of problematic behaviors	13 (2.2)	106 (5.5)	57 (7.4)	46 (12.5)	93 (16.1)		
Learning problems, n (%)							< 0.001	< 0.001
	No symptoms	585 (97.2)	1831 (95.4)	715 (92.4)	328 (88.9)	494 (85.3)		
	Symptoms of problematic behaviors	17 (2.8)	88 (4.6)	59 (7.6)	41 (11.1)	85 (14.7)		
Psychosomatic problems, n (%)							< 0.001	< 0.001
	No symptoms	583 (96.8)	1815 (94.6)	701 (90.6)	330 (89.4)	503 (86.9)		
	Symptoms of problematic behaviors	19 (3.2)	104 (5.4)	73 (9.4)	39 (10.6)	76 (13.1)		
Impulsive-hyperactive, n (%)							< 0.001	< 0.001
	No symptoms	584 (97.0)	1831 (95.4)	724 (93.5)	328 (88.9)	491 (84.8)		
	Symptoms of problematic behaviors	18 (3.0)	88 (4.6)	50 (6.5)	41 (11.1)	88 (15.2)		
Anxiety, n (%)							< 0.001	< 0.001
	No symptoms	591 (98.2)	1835 (95.6)	731 (94.4)	328 (88.9)	512 (88.4)		
	Symptoms of problematic behaviors	11 (1.8)	84 (4.4)	43 (5.6)	41 (11.1)	67 (11.6)		
Hyperactivity index, n (%)							< 0.001	< 0.001
	No symptoms	580 (96.3)	1806 (94.1)	709 (91.6)	318 (86.2)	473 (81.7)		
	Symptoms of problematic behaviors	22 (3.7)	113 (5.9)	65 (8.4)	51 (13.8)	106 (18.3)		

*ACEs* adverse childhood experiences, *SD* standard deviation

^a^ i.e., single, divorced, separated, and widowed

^b^ 1 US $ ≈ 6.92 RMB

The association between maternal ACEs and children’s behavioral problems was presented in Table 2. In the crude model, children of mothers with one or more ACE exposure had significantly higher risk of conduct problems, psychosomatic problems, anxiety, and hyperactivity index, compared to those without maternal ACE exposure. However, only children with two or more maternal ACEs were at increased risk of learning problems and impulsive-hyperactive. As the number of maternal ACEs increased, the risk of children’s behavioral problems also increased across all dimensions (all *p* values for trend < 0.001). After adjustment for covariates, the association between maternal ACEs and different types of behavioral problems were weakened, but remained significant with dose-response patterns. Specifically, children of mothers exposed to four or more ACEs had 4.99 (95%CI: 2.69 to 9.26), 4.23 (95%CI: 2.43 to 7.35), 3.25 (95%CI: 1.89 to 5.57), 3.99 (95%CI: 2.32 to 6.86), 3.52 (95%CI: 1.77 to 7.00), and 3.71 (95%CI: 2.25 to 6.11) times increased odds of conduct problems, learning problems, psychosomatic problems, impulsive-hyperactive, anxiety, and hyperactivity index, respectively.

**Table 2:** Association between maternal ACEs and children’s behavioral problems

Behavioral problems	OR (95%CI) by number of ACEs					*p*-value for trend
	0	1	2	3	≥4	
	Crude model					
Conduct problems	Ref	2.65 (1.48, 4.75) *	3.60 (1.95, 6.64) *	6.45 (3.44, 12.12) *	8.67 (4.79, 15.68) *	< 0.001
Learning problems	Ref	1.65 (0.98, 2.80)	2.84 (1.64, 4.92) *	4.30 (2.41, 7.69) *	5.92 (3.47, 10.10) *	< 0.001
Psychosomatic problems	Ref	1.76 (1.07, 2.89) *	3.20 (1.91, 5.36) *	3.63 (2.06, 6.38) *	4.64 (2.77, 7.77) *	< 0.001
Impulsive-hyperactive	Ref	1.56 (0.93, 2.61)	2.24 (1.29, 3.88) *	4.06 (2.29, 7.18) *	5.82 (3.45, 9.79) *	< 0.001
Anxiety	Ref	2.46 (1.30, 4.64) *	3.16 (1.62, 6.18) *	6.72 (3.41, 13.25) *	7.03 (3.68, 13.45) *	< 0.001
Hyperactivity index	Ref	1.65 (1.04, 2.63) *	2.42 (1.47, 3.97) *	4.23 (2.52, 7.10) *	5.91 (3.67, 9.50) *	< 0.001
	Adjusted model^a^					
Conduct problems	Ref	2.08 (1.15, 3.73) *	2.69 (1.45, 5.01) *	4.25 (2.21, 8.16) *	4.99 (2.69, 9.26) *	< 0.001
Learning problems	Ref	1.40 (0.82, 2.38)	2.39 (1.37, 4.18) *	3.31 (1.82, 6.00) *	4.23 (2.43, 7.35) *	< 0.001
Psychosomatic problems	Ref	1.59 (0.97, 2.63)	2.83 (1.69, 4.75) *	2.82 (1.59, 4.99) *	3.25 (1.89, 5.57) *	< 0.001
Impulsive-hyperactive	Ref	1.28 (0.76, 2.16)	1.82 (1.04, 3.18) *	2.89 (1.61, 5.20) *	3.99 (2.32, 6.86) *	< 0.001
Anxiety	Ref	2.01 (1.05, 3.82) *	2.41 (1.22, 4.79) *	4.17 (2.06, 8.44) *	3.52 (1.77, 7.00) *	< 0.001
Hyperactivity index	Ref	1.31 (0.82, 2.11)	1.88 (1.13, 3.12) *	2.95 (1.72, 5.05) *	3.71 (2.25, 6.11) *	< 0.001

*ACEs* adverse childhood experiences

^a^Adjusted models were controlled for maternal age, current marital status, educational level, monthly per-capita income, negative emotional states, as well as children’s age, gender, single-child status, and primary caregivers

*p-value < 0.05

**Fig. 1:**
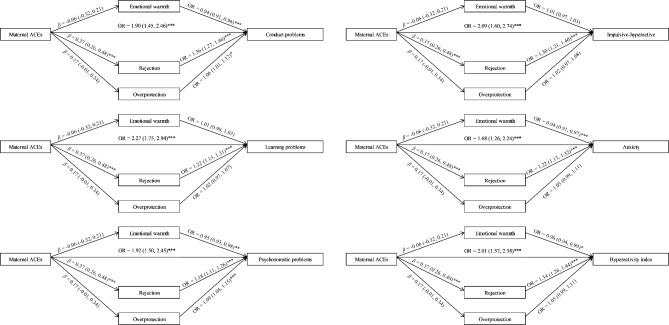
The mediation effects of maternal parenting styles in the association between maternal ACEs and children’s behavioral problems *ACEs* adverse childhood experiences *p < 0.05, **p < 0.01, ***p < 0.001

Since maternal exposure to two or more ACEs was significantly associated with children’s behavioral problems across all dimensions, we converted maternal ACE scores into a binary variable for mediation analyses, i.e., < 2 ACEs and ≥ 2 ACEs. The mediating effect of maternal parenting styles was presented in Table 3 and Fig. 1. After adjustment for covariates, maternal exposure to ≥ 2 ACEs was significantly associated with parenting styles of rejection (*β* = 0.37, 95%CI: 0.26 to 0.48). However, the association of maternal ACEs with emotional warmth and overprotection was not statistically significant. In addition, maternal parenting style of rejection was positively associated with all dimensions of behavioral problems (OR = 1.36, 95%CI: 1.27 to 1.46 for conduct problems, OR = 1.22, 95%CI: 1.15 to 1.31 for learning problems, OR = 1.18, 95%CI: 1.11 to 1.26 for psychosomatic problems, OR = 1.30, 95%CI: 1.21 to 1.40 for impulsive-hyperactive, OR = 1.22, 95%CI: 1.13 to 1.32 for anxiety, and OR = 1.34, 95%CI: 1.26 to 1.44 for hyperactivity index), while overprotection was associated with increased odds of conduct problems (OR = 1.06, 95%CI: 1.01 to 1.12) and psychosomatic problems (OR = 1.09, 95%CI: 1.04 to 1.15). In the contrary, emotional warmth was negatively associated with children’s conduct problems (OR = 0.94, 95%CI: 0.91 to 0.96), psychosomatic problems (OR = 0.95, 95%CI: 0.93 to 0.98), anxiety (OR = 0.94, 95%CI: 0.91 to 0.97), and hyperactivity index (OR = 0.96, 95%CI: 0.94 to 0.99).

Furthermore, maternal parenting styles of rejection was a significant mediator in the association of maternal ACEs and children’s behavioral problems (OR = 1.12, 95%CI: 1.08 to 1.17 for conduct problems, OR = 1.08, 95%CI: 1.05 to 1.12 for learning problems, OR = 1.06, 95%CI: 1.04 to 1.10 for psychosomatic problems, OR = 1.10, 95%CI: 1.06 to 1.15 for impulsive-hyperactive, OR = 1.08, 95%CI: 1.04 to 1.12 for anxiety, and OR = 1.12, 95%CI: 1.08 to 1.17 for hyperactivity index). The proportion of mediation ranged from 8.4% for both learning problems and psychosomatic problems to 15.0% for conduct problems. Neither emotional warmth nor overprotection significantly mediated the association between maternal ACEs and children’s behavioral problems.

**Table 3:** The mediation effects of maternal parenting styles in the association between maternal ACEs and children’s behavioral problems

	OR (95% CI) by maternal ACEs^a^					Proportion of mediation^b^
	Direct effect	Indirect effect via parenting styles			Total effect	
		Emotional warmth	Rejection	Overprotection		
Conduct problems	1.90 (1.49, 2.47) *	1.00 (0.99, 1.03)	1.12 (1.08, 1.17) *	1.01 (1.00, 1.03)	2.16 (1.67, 2.82) *	15.0%
Learning problems	2.27 (1.72, 2.98) *	1.00 (0.99, 1.00)	1.08 (1.05, 1.12) *	1.00 (1.00, 1.02)	2.46 (1.85, 3.22) *	8.4%
Psychosomatic problems	1.92 (1.50, 2.43) *	1.00 (0.99, 1.02)	1.06 (1.04, 1.10) *	1.01 (1.00, 1.04)	2.07 (1.64, 2.64) *	8.4%
Impulsive-hyperactive	2.09 (1.57, 2.72) *	1.00 (0.99, 1.01)	1.10 (1.06, 1.15) *	1.00 (1.00, 1.02)	2.32 (1.74, 2.98) *	11.6%
Anxiety	1.68 (1.26, 2.27) *	1.00 (0.99, 1.02)	1.08 (1.04, 1.12) *	1.01 (1.00, 1.03)	1.83 (1.39, 2.47) *	12.3%
Hyperactivity index	2.01 (1.58, 2.57) *	1.00 (0.99, 1.02)	1.12 (1.08, 1.17) *	1.01 (1.00, 1.02)	2.27 (1.77, 2.91) *	13.4%

*ACEs* adverse childhood experiences

^a^Models were controlled for maternal age, current marital status, educational level, monthly per-capita income, negative emotional states, as well as children’s age, gender, single-child status, and primary caregivers

^b^Proportion of mediation was calculated as the indirect effect via parenting styles divided by the total effect if the indirect effect was statistically significant

*p-value < 0.05

## Discussion

In this cross-sectional study, we found a significant association between maternal exposure to ACEs and preschool children’s behavioral problems. Dose-response patterns were also observed in the associations. In addition, maternal parenting styles of rejection was a significant mediator, accounting for approximately 8.4–15.0% of the associations. Neither maternal emotional warmth nor overprotection emerged as significant mediators in the association of maternal ACE exposure with behavioral problems in their preschool offspring.

Our findings revealed a dose-response association between maternal exposure to ACEs and the risk of behavioral problems in their children, which aligned with previous studies [11, 33–35]. For example, a cross-sectional study of 7318 preschool children has reported a significant link between maternal ACEs and children’s behavioral problems (aOR = 2.91, 95% CI: 2.45–3.45) [11]. A prospective study that followed 1994 mothers from pregnancy until their child reached 3 years of age has shown that children of mothers with ACE exposure were at a higher risk of both internalizing and externalizing behaviors [33]. Another cohort study of 1030 mother-child dyads has found a small yet significant association of maternal ACEs with internalizing behaviors in the next generation aged between 4 and 6 years [34]. A systematic review including 11 relevant studies has also illustrated that maternal ACEs was significantly associated with children’s externalizing behavioral problems, such as inattention, hyperactivity, impulsivity, and aggression [35].

Although the exact mechanisms underlying the association between maternal ACEs and children’s behavioral problems were unclear, several possible explanations might account for such associations. First, experience of childhood adversity is known to generate chronic stress, which could result in long-term alterations in stress response systems, including heightened hypothalamic-pituitary-adrenal (HPA) axis reactivity [36]. When women with ACEs become pregnant, these alterations, such as elevated concentration of stress hormones, may affect fetal development through impaired placental functioning and unfavorable uterine environment [37]. In addition, stress stemming from childhood adversity is associated with risky health behaviors during pregnancy, including smoking, alcohol use, and illicit drug use [38]. All these prenatal and perinatal factors have been demonstrated to be associated with the development of offspring behavioral problems later in life [36, 39, 40]. Second, experiencing ACEs might lead to negative emotions in mothers, such as depression and anxiety [8, 41], which has been recognized as significant risk factors for behavioral problems in their children [42]. In addition, these negative emotions could impede a mother’s ability to provide responsive and sensitive care to her children [43], thereby increasing the risk of behavioral problems in their children [44]. Third, previous research has demonstrated that ACEs were associated with low socioeconomic status, such as low educational attainment, unemployment, and limited financial income [45]. These conditions may aggravate the parent-child relationship and diminish the quality of the family environment, subsequently contributing to the development of children’s behavioral problems [46].

We have also found that maternal parenting styles of rejection was a significant mediator in the association between maternal ACEs and children’s behavioral problems, which was in line with previous studies [20, 34]. For example, a follow-up study of 488 mother and their children has indicated that mothers who were exposed to ACEs were more likely to report parenting stress and employ punitive and aggressive parenting behaviors, which were significantly linked to children’s externalizing behaviors [20]. Another longitudinal study has found that mothers with ACE exposure struggled to provide positive parenting behaviors for their children, which in turn was associated with children’s internalizing behaviors [34]. However, a prospective study of 490 mother-child dyads has found that maternal responsive parenting behaviors were not significant mediators in the association between maternal ACEs of abuse and children’s internalizing problems [47]. Another US-based cohort study has even shown that maternal ACEs of maltreatment were not significant predictors of parenting quality [21]. The discrepancies in findings could be attributed to variations in parenting styles measured and different ACE indicators collected across investigations [24]. In addition, previous studies have suggested that maternal mental health was a proximal mediator in the association of maternal ACEs and children’s outcomes [47, 48]. Our findings confirmed the mediating role of parenting styles after adjustment for maternal negative emotional states, emphasizing the independent impacts of ACE exposure on parenting practices and the importance of family-level mediators.

Our study utilized a sufficient number of mother-child dyads, which reinforced the statistical power. We further investigated the mediating role of parenting styles in the association between maternal ACEs and children’s behavioral problems. As mothers play crucial roles in shaping young children’s growth and development, our findings offer importance intervention implications. Nevertheless, several drawbacks of this study deserve further discussion. First, this study has a cross-sectional design. Previous evidence suggests that the association between parental styles and children’s behavioral problems was bidirectional [16]. Future longitudinal studies are necessary to further investigate the temporal link of maternal ACEs, parenting styles, and children’s behavioral problems. Second, although we have applied a multistage sampling strategy, all mother-child dyads were recruited from a single megacity in southwest China. The generalizability to other parts of China, particularly the rural areas, should be further confirmed. Third, maternal ACEs were collected based on retrospective measurements, which were subject to recall bias. Even though previous research has proven the reliability of retrospective ACE data [49], future investigation should further confirm whether these associations were present between prospective measured ACEs and children’s health outcomes. Fourth, although several established confounders have been adjusted in multivariate data analyses, we cannot rule out the presence of residual confounders, which might influence the observed association [30, 50, 51]. In addition, previous studies have demonstrated that neurological problems and physical complaints were linked to behavioral problems in children [52, 53]. Nevertheless, due to data unavailability, we were unable to examine the potential impact of these factors on the observed association.

## Conclusions

In conclusion, this cross-sectional study showed that maternal ACEs were significantly associated with behavioral outcomes in Chinese preschool children. The results further support the mediating role of maternal parenting styles of rejection in this association. From one side, our study underscores the importance of identifying mothers with a history of ACEs and providing timely and appropriate support to their children, which might reduce the risk of behavioral problems in the offspring. From the other side, interventions targeting at improving parenting skills and fostering secure attachment might be an effective strategy to mitigate the detrimental consequences of maternal ACEs on children’s behaviors. Nevertheless, further randomized controlled trials are needed to confirm the conclusions and elucidate the possible mechanism underlying the intergenerational transmission of ACEs.

### Electronic supplementary material

Below is the link to the electronic supplementary material.

Supplementary Material 1

## Data Availability

The datasets used and analyzed during the current study are not publicly available for ethical and privacy reasons but are available from the corresponding author upon reasonable request.

## Abbreviations

ACEs: Adverse childhood experiences
ACE-IQ: Adverse Childhood Experiences-International Questionnaire
S-EMBU-P: Short Egna Minnen Beträffande Uppfostran Parent Form
CPRS-48: 48-item Conners’ Parent Rating Scale
DASS-21: Depression Anxiety Stress Scale-21
SD: Standard deviation
ANOVA: Analysis of Variance
OR: Odds ratios
CI: Confidence interval
GSEM: Generalized structural equation models
HPA: Hypothalamic-pituitary-adrenal
