# Supplementary material for: Maternal adverse childhood experiences and behavioral problems in preschool offspring: the mediation role of parenting styles
Source: Child Adolesc Psychiatry Ment Health. 2023 Aug 10;17:95. doi: 10.1186/s13034-023-00646-3 (PMC10416370; doi:10.1186/s13034-023-00646-3)
Supplement: Supplementary file 1 — Supplementary Material 1 [file 13034_2023_646_MOESM1_ESM.docx]

**Maternal adverse childhood experiences and behavioral problems in preschool offspring: the mediation role of parenting styles**

*Child and Adolescent Psychiatry and Mental Health*

Shengyu Luo^1^, Dezhong Chen^1^, Chunrong Li^2, 3^, Li Lin^1^, Weiqing Chen^1^, Yan Ren^2^, Yuchi Zhang^3^, Fenglin Xing^4^, Vivian Yawei Guo^1*^

^1^Department of Epidemiology, School of Public Health, Sun Yat-sen University, Guangzhou, Guangdong, China

^2^Chengdu Women’s and Children’s Central Hospital, School of Medicine, University of Electronic Science and Technology of China, Chengdu, Sichuan, China

^3^Chengdu Jintang County maternal and Child Health Hospital, Chengdu, Sichuan, China

^4^Chengdu Qingyang District maternal and Child Health Hospital, Chengdu, Sichuan, China

^*^Correspondence: Vivian Yawei Guo, Email: [guoyw23@mail.sysu.edu.cn](mailto:guoyw23@mail.sysu.edu.cn)

**Table S1** ACE items and definitions of positive answers

| **ACE categories** | **Items (While you were growing up, before the age of 18 …)** |
| --- | --- |
| Physical abuse | Did a parent, guardian or other household member spank, slap, kick, punch or beat you up? (Never, once*, a few times*, or many times*) |
|  | Did a parent, guardian or other household member hit or cut you with an object, such as a stick (or cane), bottle, club, knife, whip etc? (Never, once*, a few times*, or many times*) |
| Emotional abuse | Did a parent, guardian or other household member yell, scream or swear at you, insult or humiliate you? (Never, once*, a few times*, or many times*) |
|  | Did a parent, guardian or other household member threaten to, or actually, abandon you or throw you out of the house? (Never, once*, a few times*, or many times*) |
| Physical neglect | How often did your parents/guardians not give you enough food even when they could easily have done so? (Never, once*, a few times*, or many times*) |
|  | Were your parents/guardians too drunk or intoxicated by drugs to take care of you? (Never, once*, a few times*, or many times*) |
|  | How often did your parents/guardians not send you to school even when it was available? (Never, once*, a few times*, or many times*) |
| Emotional neglect | Did your parents/guardians understand your problems and worries? (Never*, rarely*, sometimes*, most of time, or always) |
|  | Did your parents/guardians really know what you were doing with your free time when you were not at school or work? (Never*, rarely*, sometimes*, most of time, or always) |
| Witnessing domestic violence | Did you see or hear your father/mother being yelled at, screamed at, sworn at, insulted or humiliated by your mother/father? (Never, once*, a few times*, or many times*) |
|  | Did you see or hear your father/mother being slapped, kicked, punched or beaten up by your mother/father? (Never, once*, a few times*, or many times*) |
|  | Did you see or hear your father/mother being hit or cut with an object, such as a stick (or cane), bottle, club, knife, whip etc., by your mother/father? (Never, once*, a few times*, or many times*) |
| Household substance abuse | Did you live with a household member who was a problem drinker or alcoholic, or misused street or prescription drugs? (Yes* or no) |
| Incarcerated household member | Did you live with a household member who was ever sent to jail or prison? (Yes* or no) |
| Household mental illness | Did you live with a household member who was depressed, mentally ill or suicidal? (Yes* or no) |
| Parental divorce or separation | Were your parents ever separated or divorced? (Yes* or no) |
| Parental death | Did your mother, father or guardian die? (Yes* or no) |
| Bullying | How often were you bullied? (Never, once*, a few times*, or many times*) |
| Community violence | Did you see or hear someone being beaten up in real life? (Never, once*, a few times*, or many times*) |
|  | Did you see or hear someone being stabbed or shot in real life? (Never, once*, a few times*, or many times*) |
|  | Did you see or hear someone being threatened with a knife or gun in real life? (Never, once*, a few times*, or many times*) |

* Indicates positive answers for ACE items

*ACEs* adverse childhood experiences
